# The incidence of acute pancreatitis: impact of social deprivation, alcohol consumption, seasonal and demographic factors^1^

**DOI:** 10.1111/apt.12408

**Published:** 2013-07-16

**Authors:** SE Roberts, A Akbari, K Thorne, M Atkinson, PA Evans

**Affiliations:** * College of Medicine, Swansea UniversitySwansea, UK; † Department of Emergency Medicine, Morriston HospitalSwansea, UK

## Abstract

**Background:**

The incidence of acute pancreatitis has increased sharply in many European countries and the USA in recent years.

**Aim:**

To establish trends in incidence and mortality for acute pancreatitis in Wales, UK, and to assess how incidence may be linked to factors including social deprivation, seasonal effects and alcohol consumption.

**Methods:**

Use of record linked inpatient, mortality and primary care data for 10 589 hospitalised cases of acute pancreatitis between 1999 and 2010.

**Results:**

The incidence of acute pancreatitis was 30.0 per 100 000 population overall, mortality was 6.4% at 60 days. Incidence increased significantly from 27.6 per 100 000 in 1999 to 36.4 in 2010 (average annual increase = 2.7% per year), there was little trend in mortality (0.2% average annual reduction). The largest increases in incidence were among women aged <35 years (7.9% per year) and men aged 35–44 (5.7%) and 45–54 (5.3%). Incidence was 1.9 times higher among the most deprived quintile of patients compared with the most affluent (3.9 times higher for alcoholic acute pancreatitis and 1.5 for gallstone acute pancreatitis). Acute pancreatitis was increased significantly during the Christmas and New Year weeks by 48% (95% CI = 24–77%) for alcoholic aetiology, but not for gallstone aetiology (9%). Alcoholic admissions were increased with higher consumption of spirits and beer, but not wine.

**Conclusions:**

The study shows an elevated rate of alcoholic acute pancreatitis during the Christmas and New Year period. Acute pancreatitis continues to rise, most rapidly for young women, while alcoholic acute pancreatitis is linked strongly with social deprivation.

## Introduction

There have been sharp increases in the incidence of acute pancreatitis over the last 30 years in the UK,^1^–^6^ and in many, but not all, studies of other European or western countries. These include studies in Croatia,^7^ Denmark,^8^ Finland,^9^,^10^ Germany,^11^ Ireland,^12^ Norway,^13^ Sweden,^14^–^16^ the Netherlands,^17^,^18^ and the USA.^19^,^20^

Although several studies have investigated the incidence of acute pancreatitis according to the month of the year,^21^–^23^ none have reported as to whether it is increased according to seasonal factors such as Christmas and the New Year. Little has also been reported as to how acute pancreatitis is related to socio-economic status,^5^,^24^,^25^ while evidence about the relationship between alcohol consumption and acute pancreatitis is complex and sometimes conflicting.^26^–^32^

The main objectives of this study were, firstly, to establish trends in the incidence and subsequent mortality for acute pancreatitis over the recent 12-year period from 1999 to 2010 in Wales, UK. Secondly, to assess how the incidence of acute pancreatitis may be linked to factors including social deprivation, seasonal effects, alcohol consumption and demography.

The main study hypotheses are that the incidence of acute pancreatitis has increased over time, is increased during the Christmas holiday period, and is linked strongly with social deprivation.

## Methods

To investigate acute pancreatitis across Wales, UK (population 3.0 million), we used systematic record linkage of national in-patient, mortality and primary care data. These data are incorporated along with other health and social services data in the Secure Anonymised Information Linkage (SAIL) Databank,^33^,^34^ funded by the Welsh Assembly Government since 2006 and stored on an IBM supercomputer in the College of Medicine, Swansea University. These data have been used as the basis of many published studies in international Medline journals.^35^–^40^ The hospital in-patient data used, the Patient Episode Database for Wales (PEDW), includes abstracts of in-patient and day case admissions to all National Health Service (NHS) hospitals in Wales. The in-patient data were systematically linked together to enable subsequent admissions for the same people to be traced. They were then record linked to mortality data from the Office for National Statistics (ONS) and the NHS Welsh Administrative Register to identify deaths that occurred following discharge from hospital along with the in-patient deaths. The in-patient and mortality data were also record linked to SAIL primary care data, obtained from 35% of all general practices across Wales during the entire study period (population 1.0 million) to obtain further information on aetiology. The record linkage of these in-patient, mortality and primary care data has been validated previously and has been shown to be >99.8% accurate.^34^

### Study inclusion and exclusion criteria

The study included admissions for acute pancreatitis during the 12-year period from 1 January 1999 to 31 December 2010, with 60 day follow-up to 1 March 2011. We selected only those admissions where acute pancreatitis was recorded as the principal diagnosis on the discharge record and included all sources of admission. The International Classification of Diseases tenth revision (ICD-10) code used for acute pancreatitis was K85. We included each person's first admission for acute pancreatitis after the start of the study period on 1 January 1999. As acute pancreatitis is often characterised by subsequent attacks that each require hospitalisation, we included subsequent admissions for acute pancreatitis for each individual – as subsequent attacks – if they occurred at least 60 days following discharge from a previous admission.

### Aetiology of acute pancreatitis

We determined the two main aetiologies of acute pancreatitis (gallstone or biliary and alcohol) as follows. Firstly, gallstone acute pancreatitis was defined where there was a diagnosis of cholelithiasis (ICD-10 code = K80) or cholecystitis (K81) recorded in any diagnostic position on the in-patient record during the patient's current admission, previous admissions or from primary care consultations during the previous 5 years. Gallstone acute pancreatitis was also determined if the following surgical procedures were recorded in any position during the current or previous in-patient admissions during the last 5 years: total cholecystectomy and excision of surrounding tissue (OPCS-4 procedure code = J18.1), endoscopic sphincterotomy of sphincter of odi and removal of calculus HFQ (J38.1) or endoscopic retrograde extraction of calculus from bile duct (J41.1). Alcoholic pancreatitis was defined where any one of 21 alcohol-attributable diagnoses or symptoms were similarly recorded in any position during the current admission, previous admissions or primary care consultations. These 21 conditions and their ICD-10 codes are listed in Appendix1. Other aetiologies of acute pancreatitis were similarly defined from diagnoses recorded in any position on the current or previous health records; including hyperlipidaemia (ICD-10 code = E78), hypercalcaemia (E83.5), malnutrition (E40–E46), abdominal trauma (S30–S39), pancreatic malignancies (C25) and cystic fibrosis (E84).

### Exposure measures

We measured social deprivation using the Welsh Index of Multiple Deprivation (WIMD), 2005 version as this was at the midpoint of the study period from 1999 to 2010.^41^ WIMD is similar to the widely used English Indices of Multiple Deprivation (IMD),^42^ and WIMD 2005 comprises seven separate domains of deprivation. These are ‘income’ (25% contribution), ‘employment’ (25%), ‘education’ (15%), ‘health’ (15%), ‘geographical access to services’ (10%), ‘housing’ (5%) and ‘physical environment’ (5%). The total WIMD deprivation scores were assigned anonymously to 1896 Lower Super Output Areas (LSOAs) across Wales (average LSOA population = 1560). The LSOAs were then ranked according to their social deprivation score and were categorised into quintiles (I = least deprived and V = most deprived quintile).

Information on the consumption of different types of alcoholic beverage were obtained from the official Welsh Health Survey, collected during the three survey years from 2008 to 2010.^43^ The Welsh Health Survey is a nationally representative sample of approximately 15 000 adults (0.5% of the population) each year. Respondents reported the numbers of units of wine, beer and spirits that they had consumed on the day in the week prior to the survey on which they had consumed the most alcohol, which were then standardised using the resident population of Wales. The mean alcohol consumption figures were then correlated with acute pancreatitis incidence across the 94 study Upper Super Output Areas (USOAs; average USOA population = 32 000 and average number of survey respondents per USOA each year = 160) during the same 3-year period from 2008 to 2010.

We also compared the incidence of acute pancreatitis for admissions on weekends (00:00 hours on Saturday to 00:00 hours on Monday) on weekdays and on bank holidays. Seasonal effects in the incidence of acute pancreatitis were assessed according to the calendar month of each admission and according to daily admissions in December and January, and weekly admissions during the Christmas and New Year weeks (last week of December and first week of January).

### Outcome measures and methods of analysis

The main study outcome measures were incidence rates for acute pancreatitis per 100 000 population and mortality rates at 60 days following admission. Although mortality at 30 days is also reported, 60 days was chosen as the preferred mortality outcome measure as a 30 day limit would exclude some deaths that occur during prolonged in-patient stays for severe necrotising cases.^5^ Incidence rates were calculated using the numbers of hospitalised cases for acute pancreatitis as numerators and the corresponding resident populations as denominators. The incidence rates were then standardised using the direct method and the Welsh resident population during the study period as the standard, and were expressed per 100 000 population. Percentage mortality was calculated by dividing the numbers of deaths (from all causes) by the numbers of hospitalised cases and was standardised directly using the study population hospitalised with acute pancreatitis. Other methods of analysis include mean annual changes over time in incidence and mortality rates, relative risks and their 95% CIs to compare seasonal incidence rates, Spearman's rank correlations to assess possible links between incidence and alcohol consumption, one way analysis of variance (anova) to assess trends over time in durations of in-patient stay, and weighted 5 year moving averages to smooth daily trends in incidence. Significance was measured at the conventional 5% level.

## Results

During the 12-year study period, there were a total of 10 589 separate attacks of acute pancreatitis, among 8607 different patients. 7356 of the patients (85.5%) were admitted once only during the study period, 848 (9.9%) were admitted twice, 242 (2.8%) three times and 161 (1.9%) four times or more. The mean age of the patients was 57.7 years (s.d. = 19.2) and a slight majority (4362; 50.7%) were men. The overall incidence of acute pancreatitis, based on the 10 589 cases, was 30.0 per 100 000 population and the mortality rates at 30 days and 60 days were 5.6% and 6.4% respectively (based on 560 and 675 deaths).

Of the 10 589 cases, 3903 (36.9%) were of gallstone aetiology and 2327 (22.0%) were alcohol-induced. Other aetiologies or diagnoses recorded include hyperlipidaemia (1068; 10.1%), abdominal trauma (161; 1.5%), hypercalcaemia (61; 0.6%), malnutrition (27; 0.3%), pancreatic malignancies (21; 0.2%) and cystic fibrosis (12; 0.1%).

**Figure 1:**
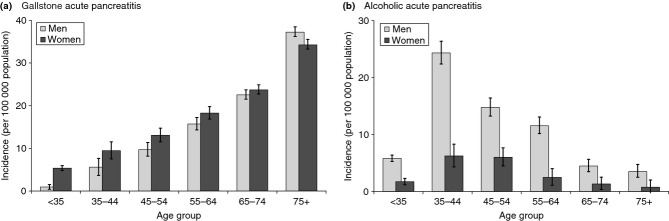
Incidence of acute pancreatitis (per 100 000 population) for men and women in different age groups for gallstone and alcohol aetiologies, in Wales from 1999 to 2010. (a) Gallstone acute pancreatitis. (b) Alcoholic acute pancreatitis. Vertical bars represent 95% confidence intervals.

The incidence of acute pancreatitis overall was significantly higher (*P* < 0.001) among men (32.8 per 100 000) than women (27.8) and it increased across age groups (Table 1). The incidence of acute pancreatitis of gallstone aetiology increased sharply with age for both men and women (Figure1a,b). It was significantly higher among women than men in younger age groups (<55 years) but more similar in men and women in older age groups. Alcoholic acute pancreatitis occurred most frequently among the 34–44 and 45–54 year age groups in both men and women, and was significantly higher among men than women in all age groups (Table 1).

**Table 1:** Number of cases of acute pancreatitis, aetiology and incidence rates (per 100 000 population) with corresponding average annual percentage increases, according to age group and gender, in Wales from 1999 to 2010

	Number of cases of acute pancreatitis	% of cases of gallstone aetiology	% of cases of alcohol aetiology	Incidence per 100,000 population (95% CI)	Average annual change in incidence (%)
Men					
<35	844	8.4	51.5	11.3 (10.5, 12.1)	2.9
35–44	958	14.0	60.9	40.0 (35.2, 44.8)	5.7
45–54	995	22.5	43.3	43.1 (39.1, 47.1)	5.3
55–64	965	35.2	25.9	44.6 (39.5, 49.7)	4.8
65–74	898	39.3	7.8	57.3 (52.5, 62.1)	3.9
75+	925	46.0	4.3	80.8 (72.8, 88.8)	2.6
All ages	5585	27.7	32.4	32.8 (29.9, 35.7)	3.4
Women					
<35	763	51.0	16.4	10.5 (9.1, 11.9)	7.9
35–44	563	38.2	27.9	22.4 (20.1, 24.7)	0.6
45–54	692	45.2	20.8	28.9 (26.3, 31.5)	4.2
55–64	867	46.6	6.3	39.2 (36.6, 41.8)	−0.5
65–74	802	51.0	2.9	46.5 (43.8, 49.2)	−0.6
75+	1316	47.5	1.1	71.0 (66.5, 75.5)	3.4
All ages	5003	47.1	10.4	27.8 (26.4, 29.2)	1.9
All patients	10 589	36.9	22.0	30.0 (27.8, 32.0)	2.7

The gender of one patient was not recorded.

### Trends in acute pancreatitis

**Figure 2:**
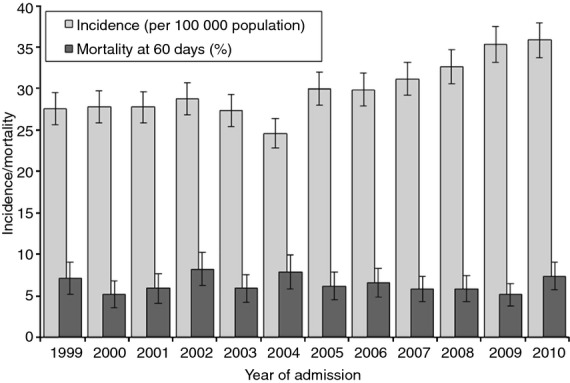
Trends in incidence (per 100 000 population) and mortality (at 60 days) following admission for acute pancreatitis, in Wales from 1999 to 2010. Incidence and mortality are standardised for age group and gender. Vertical bars represent 95% confidence intervals.

The incidence of acute pancreatitis increased from 27.6 per 100 000 population in 1999 to 35.9 per 100 000 in 2010 (Figure2) with a significant (*P* < 0.001) mean annual increase of 2.7% per annum over the 12-year period (Table 1). There was little trend over time in mortality (mean annual reduction = 0.2%; Figure2). Of all demographic age groups, incidence rose most sharply for women aged <35 years (7.9% increase per year), followed by men aged 35–44 years (5.7%) and men aged 45–54 years (5.3%; Table 1). Alcoholic acute pancreatitis rather than gallstone acute pancreatitis was the dominant aetiology in these three age-gender groups (43.2% vs. 27.4%; *P* < 0.001) while gallstone acute pancreatitis was more common in all other age-gender groups (40.5% vs. 13.8%; *P* < 0.001). The median length of stay was 6 days overall and fell over time from 7 days in 1999, 6 days during every year from 2000 to 2007 and 5 days in 2008, 2009 and 2010 (*P* < 0.001).

### Acute pancreatitis and social deprivation

**Figure 3:**
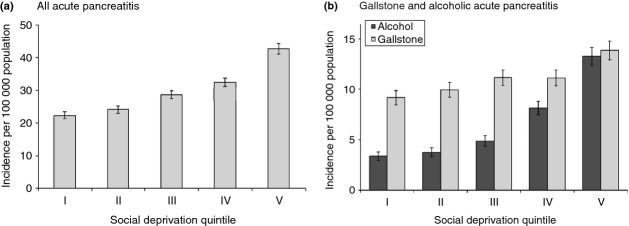
Incidence of acute pancreatitis (per 100 000 population) according to social deprivation quintile (I = most affluent, V = most deprived) in Wales from 1999 to 2010 for (a) All acute pancreatitis. (b) Gallstone and alcoholic acute pancreatitis. Incidence is standardised for age group and gender. Vertical bars represent 95% confidence intervals.

The incidence of acute pancreatitis was 1.9 times higher (95% CI = 1.8–2.0) among the most deprived quintile of patients compared with the most affluent quintile (Figure3a). Figure3b shows that this association between acute pancreatitis and social deprivation was much stronger for alcoholic aetiology (3.9; 95% CI = 3.4–4.5) than for gallstone aetiology (1.5; 1.4–1.7), or for all other and unspecified aetiologies (1.6; 95% CI = 1.4–1.7).

### Acute pancreatitis and alcohol consumption

Across the 94 USOAs in Wales, there were positive correlations between the incidence of alcoholic acute pancreatitis and the average reported unit consumption of beer (0.46; *P* < 0.001), spirits (0.30; *P* = 0.004) and total alcohol (beer, spirits and wine; 0.35; *P* = 0.001) but there was a negative correlation between alcoholic acute pancreatitis and wine (−0.37; *P* < 0.001). Consumption of beer was correlated positively with spirits (0.22; *P* = 0.03) and negatively with wine (−0.58; *P* < 0.001) with no link between spirits and wine (0.02; *P* = 0.86). Social deprivation was correlated positively with consumption of beer (0.38; *P* < 0.001) and with total alcohol consumption (0.22; *P* = 0.033), negatively with wine (−0.35; *P* = 0.001) but was not correlated with spirits (0.03; *P* = 0.81).

### Acute pancreatitis, day of week and seasonal effects

**Figure 4:**
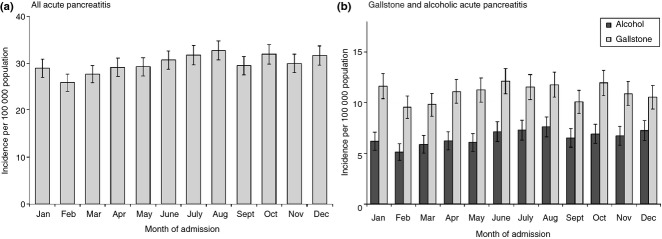
Incidence of acute pancreatitis (per 100 000 population) according to the month of the admission, in Wales from 1999 to 2010, for (a) All acute pancreatitis. (b) Gallstone and alcoholic acute pancreatitis. Incidence is standardised for age group and gender. Vertical bars represent 95% confidence intervals.

The (hospitalised) incidence for acute pancreatitis was higher on weekdays (31.8 per 100 000; 95% CI = 31.1–32.5) than on weekends (25.6; 24.7–26.6) but similar with that on bank holidays (29.2; 25.5–33.3). Incidence varied according to the calendar month (*P* < 0.001 overall, *P* = 0.009 for gallstone acute pancreatitis and *P* = 0.024 for alcoholic acute pancreatitis). Incidence was highest during the months of August (32.8 per 100 000), October (32.0), December and July (both 31.8) and was significantly higher during each of these months [all (p < 0.05) than during February or March (Figure4)].

**Figure 5:**
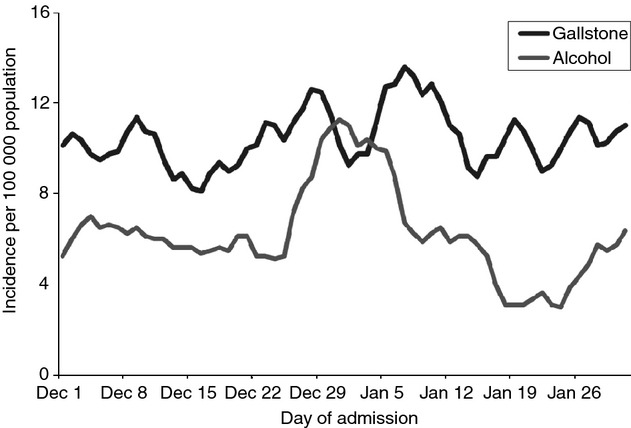
Trends in daily admission rates for gallstone and alcoholic acute pancreatitis (per 100 000 population) during the months of December and January, in Wales from 1999 to 2010.

Figure5 shows smoothed daily trends in admissions for acute pancreatitis of alcohol and gallstone aetiologies during the months of December and January. Admissions for alcoholic pancreatitis during the last week of December and the first week of January were 48% higher (24–77%) than during the rest of the year and 64% higher (95% CI = 33–103%) than during the rest of December and January. For gallstone acute pancreatitis, however, there was no significant increase (9%) during the Christmas and New Year weeks compared with either the rest of the year or the rest of December and January (12%).

## Discussion

The incidence of acute pancreatitis in this study (30 per 100 000 population) increased significantly by 2.7% per year between 1999 and 2010, but there was no trend in mortality (6.4% at 60 days). The largest increases in incidence were for young women aged <35 years and young/middle aged men aged 35–54 years, and were linked with alcoholic rather than gallstone acute pancreatitis. Acute pancreatitis was associated strongly with higher levels of social deprivation, and alcoholic acute pancreatitis was increased significantly during the Christmas and New Year weeks.

### Strengths and limitations

Major strengths of this study are, firstly, that it provides new evidence on trends in acute pancreatitis and on factors that affect its occurrence. Secondly, it is a large study, covering more than 10 000 cases of acute pancreatitis. It is based on systematic, validated record linkage of in-patient, death certificate and primary care data to identify all admissions and all deaths that occur following discharge from hospital as well as those that occur in hospital. Acute pancreatitis is also one of few disorders which almost always require hospitalisation, so that the hospitalised incidence reported in this study is a reliable measure of actual incidence.

Limitations of the study are, firstly, that the study was restricted to NHS hospitals. However, the private sector is small and receives few emergencies for acute pancreatitis. Secondly, as in other large studies of acute pancreatitis that have used administrative health data,^2^–^5^,^8^–^10^,^12^,^16^–^19^ the identification of aetiologies from recorded patient diagnoses was incomplete in some cases. Also, as with other studies based on administrative data, we included cases specifically recorded with acute pancreatitis (ICD-10 code = K85), and excluded a relatively small number of cases that were recorded vaguely on the discharge record as ‘disease of pancreas, unspecified’ (K86.9: 274 vs. 10 589 for acute pancreatitis). These cases would refer mainly to either acute pancreatitis or chronic pancreatitis and would have a very minor impact (<2.6%) on the study incidence rate. Thirdly, the administrative data also lacks detailed information about disease history, pathology, case severity and treatment. However, as this study focuses on incidence, these factors are less important. The survey data on alcohol consumption were based on self reported units drunk, which often under-reports actual consumption. Finally, we included deaths from all causes when investigating mortality, especially as acute pancreatitis was the certified underlying cause of death in only 61% of cases, which would provide an incomplete basis for ascertaining mortality.

### Trends in acute pancreatitis

During our study period from 1999 to 2010, we found a significant average annual increase of 2.7% in the incidence of acute pancreatitis. The largest increases were for young women aged under 35 years (7.9% annual increase) and young/middle aged men aged 35–54 years (5–6%), which is consistent with findings across England from 1998 to 2005.^5^ A study of Ireland from 1997 to 2004 found largest increases of acute pancreatitis overall among young men, but largest increases of alcoholic cases among young women.^12^ As alcohol rather than gallstone was the main aetiology of acute pancreatitis in these age groups, we conclude that alcohol is the main reason for the recent increases in the levels of acute pancreatitis.

### Incidence of acute pancreatitis internationally

Our incidence rate of 30 per 100 000 for acute pancreatitis in Wales is higher than 22 per 100 000 in England between 1998 and 2005, which was similarly increasing by 3% per annum.^5^ It is similar to rates reported in Scotland (32 from 1984 to 1995),^2^ Ireland (24 from 1997 to 2004),^12^ and other northern or western European countries, including Denmark (30 per 100 000 from 1981 to 2000),^8^ Iceland (32 from 1998 to 1999),^44^ Bergen, Norway (31 from 1986 to 1995),^45^ and Sweden (33 from 1998 to 2003),^16^ although higher rates of ≥40 per 100 000 have been reported previously in some of these countries. These include Buskerud County, Norway in (42 in 1992),^46^ and Stockholm County, Sweden (40 in 1974).^27^ Our incidence rate of 30 is also lower than in the USA (49 from 1988 to 2004),^47^ and in northern or eastern Europe, such as Finland (73 in 1989),^9^ Trzebnica, Poland (64 from 2005 to 2010),^48^ Świętokrzyskie Voivodeship province, Poland (100 during 2011),^49^ and north east England (57 from 2006 to 2007).^25^

To summarise, the incidence of acute pancreatitis has tended to be highest in Scandinavia and eastern Europe, along with the USA and Scotland. However, there have been reports of recent decreases or levelling off in acute pancreatitis,^10^,^11^,^27^ or alcoholic acute pancreatitis,^15^ in some northern European countries or regions, that have sometimes been linked to reductions in *per capita* alcohol consumption.^10^,^15^ In contrast, the recent increases in acute pancreatitis in our study and in England,^3^–^5^ that have been linked to alcohol,^3^,^5^ indicate that acute pancreatitis rates in England and Wales may now be approaching the previously higher rates in Scandinavia.

### Social deprivation

We found a significantly increased 1.9 fold risk of acute pancreatitis among the most deprived quintile of patients compared with the least deprived. This is slightly higher than a 1.7 increased risk nationally across England from 1998 to 2005,^5^ but lower than 2.4 in a regional study of north east of England.^25^ The increased risk for quintile V in our study was stronger for alcoholic acute pancreatitis (3.9) than for gallstone acute pancreatitis (1.5), and is broadly comparable with corresponding figures of 6.5 and 1.5 from the north east of England.^25^ However, an earlier study from the Nottingham region during the 1970s found no association with social class, but instead a link with the hardness of the drinking water.^24^

### Aetiology

We found a ratio of gallstone to alcohol aetiology of 1.7 (37 gallstone: 22 alcohol) which is lower than ratios of between 3 and 15 reported in most English studies,^5^,^6^,^24^,^50^–^52^ although other studies in England have found more equal cases of gallstone and alcoholic acute pancreatitis. For example, 30:29 in the north west Thames region,^53^ 33:20 in the Wessex region,^54^ and 43:29 in the north east of England.^25^ With increases over time in alcoholic acute pancreatitis in England, these gallstone to alcohol ratios have tended to fall in recent years. Studies of Mediterranean countries have similarly reported high ratios of >3 for gallstone to alcoholic cases; for example, Greece (74:6),^55^ Italy (49:9 and 60:13),^23^,^55^ and Croatia (60:19).^7^

The aetiology ratio in our study of 1.7 is, however, more comparable with ratios of about 1–3 reported recently in studies from the USA (33:20 and (45:45),^19^,^20^ and from northern Europe, including Scotland (42:35 and 47:33),^56^,^57^ Ireland (22:19),^12^ Germany (40:32 and 35:38),^55^,^58^ Iceland (42:32),^44^ Norway (48:19),^45^ and Sweden (42:24).^15^ Much lower ratios of gallstone to alcoholic aetiologies have been reported from eastern European countries such as Hungary (24:61),^55^ and Poland (27:49).^48^ The moderately high ratio of alcoholic to gallstone acute pancreatitis in our study probably reflects quite high levels of alcohol consumption and social deprivation in parts of Wales.^41^,^43^

### Alcohol consumption and seasonal effects

We found that alcoholic acute pancreatitis was correlated positively with reported unit consumption of beer and spirits, but negatively with wine. Two large studies from Sweden found, firstly, a dose-response association between the number of units of spirits consumed on a single occasion and the risk of acute pancreatitis, but no association for wine or beer.^28^ The second study reported a positive association between acute pancreatitis and *per capita* spirits consumption, but no association with wine or beer,^27^ while others have suggested an effect particular to the constituents in certain type of spirits.^31^,^59^ Another study reported no increased risks (for beer) in Munich during the Oktoberfest.^29^ However, evidence about the type of alcohol from other studies is sometimes inconsistent.^26^,^30^,^32^ We also found a positive association between alcoholic acute pancreatitis and the number of units of beer as well as spirits consumed, although consumption of beer was correlated significantly with consumption of spirits. We found a negative association between alcoholic acute pancreatitis and unit consumption of wine, although wine consumption was found to be associated positively with social affluence. However, as in other studies,^27^,^29^ as our alcohol data are based on self-reported consumption from survey respondents, which can be inaccurate, these findings should be regarded with some caution.

Hospitalisations for acute pancreatitis were most frequent during the months of August, October, July and December. A study of Tampere, Finland,^22^ similarly found that alcoholic acute pancreatitis peaked in July and August, followed by March, October and December, with no seasonal pattern for gallstone acute pancreatitis. These findings may indicate some type of holiday influence on acute pancreatitis. However, a study of Lüneburg County, Germany found no association with calendar month,^21^ while a study of Ferra, Italy found a modest excess from March to May.^23^

Our study shows an increased admission rate for alcoholic acute pancreatitis during the Christmas and New Year period, when there is a peak in the consumption of both alcohol and spirits. Further research should be aimed at establishing the relationship between beverage constituents and acute pancreatitis.

## Authorship

*Guarantor of the article*: S.E. Roberts and A. Akbari.

*Author contributions*: SER initiated and designed the study. SER, KT and MA reviewed the literature. AA, MA, KT and SER undertook the analyses. SER wrote the first drafts of the manuscript, all authors interpreted the study findings and contributed to subsequent drafts. All authors approved the final version of the article, including the authorship list.

## Appendix 1

### ICD-10 codes used for alcoholic acute pancreatitis aetiology: admissions with alcohol attributable diagnoses and symptoms

**Table:**

Alcohol attributable diagnoses and symptoms	ICD-10 codes
Alcohol induced chronic pancreatitis	K86.0
Alcohol-induced pseudo-Cushing's syndrome	E24.4
Mental and behavioural disorders due to use of alcohol	F10
Degeneration of nervous system due to alcohol	G31.2
Alcoholic polyneuropathy	G62.1
Alcoholic myopathy	G72.1
Alcoholic cardiomyopathy	I42.6
Alcoholic gastritis	K29.2
Alcoholic liver disease	K70
Ethanol poisoning	T51.0
Methanol poisoning	T51.1
Toxic effect of alcohol, unspecified	T51.9
Accidental poisoning by and exposure to alcohol	X45
Intentional self-poisoning by and exposure to alcohol	X65
Poisoning by and exposure to alcohol, undetermined effect	Y15
Alcohol deterrents	Y57.3
Blood alcohol level > 200 mg/L	Y90.7, Y90.8
Severe/very severe alcohol intoxication	Y91.2, Y91.3
Alcohol rehabilitation	Z50.2
Alcohol abuse counselling and surveillance	Z71.4
Problems related to lifestyle – alcohol use	Z72.1
